# When Success Is Not Enough: The Symptom Base-Rate Can Influence Judgments of Effectiveness of a Successful Treatment

**DOI:** 10.3389/fpsyg.2020.560273

**Published:** 2020-10-23

**Authors:** Fernando Blanco, María Manuela Moreno-Fernández, Helena Matute

**Affiliations:** ^1^Faculty of Psychology, University of Granada, Granada, Spain; ^2^Faculty of Psychology and Education, University of Deusto, Bilbao, Spain

**Keywords:** causal learning, cognitive bias, patients’ beliefs, base-rates, causal judgment

## Abstract

Patients’ beliefs about the effectiveness of their treatments are key to the success of any intervention. However, since these beliefs are usually formed by sequentially accumulating evidence in the form of the covariation between the treatment use and the symptoms, it is not always easy to detect when a treatment is actually working. In Experiments 1 and 2, we presented participants with a contingency learning task in which a fictitious treatment was actually effective to reduce the symptoms of fictitious patients. However, the base-rate of the symptoms was manipulated so that, for half of participants, the symptoms were very frequent before the treatment, whereas for the rest of participants, the symptoms were less frequently observed. Although the treatment was equally effective in all cases according to the objective contingency between the treatment and healings, the participants’ beliefs on the effectiveness of the treatment were influenced by the base-rate of the symptoms, so that those who observed frequent symptoms before the treatment tended to produce lower judgments of effectiveness. Experiment 3 showed that participants were probably basing their judgments on an estimate of effectiveness relative to the symptom base-rate, rather than on contingency in absolute terms. Data, materials, and R scripts to reproduce the figures are publicly available at the Open Science Framework: https://osf.io/emzbj/.

## Introduction

A great deal of health-related decisions, such as deciding whether or not to quit a treatment, or whether to replace it by an alternative option, depend on the patients’ beliefs about their symptoms and diseases, and particularly about the effectiveness of their treatments. For instance, one of the main reasons for treatment drop-out is the belief that the treatment is producing little or no observable benefit (Leventhal et al., 1992; Dilla et al., 2009). Thus, understanding how patient’s beliefs form and evolve is critical to developing strategies aimed at improving the trust and adherence to the prescribed treatments, and therefore fostering well-being among patients and users.

Previous research on experimental psychology suggests that many of these health-related decisions such as treatment adherence, or therapeutic choices, can be better understood as a result of causal learning (Rottman et al., 2017). That is, the users’ beliefs about the effectiveness of the treatment are causal in nature, i.e., “the treatment causes the symptom remission,” or “the treatment prevents me from falling ill.” Thus, it is possible to study the patients’ beliefs of treatment effectiveness through causal learning experiments (see reviews in Matute et al., 2015; Matute et al., 2019). This possibility offers a number of advantages. To begin with, we can study the formation of beliefs under highly controlled settings, by using fictitious scenarios and computerized tasks. This would be impossible in real life, in which researchers cannot manipulate parameters such as the frequency with which a treatment is used, its actual effectiveness, or the severity of symptoms. Thus, ecological studies would be limited because it is often impossible to run an experiment that unveils causal relationships between different factors and health beliefs, and most research would be limited to uncontrolled, observational studies. The second advantage of using causal learning experiments is that we can study health beliefs in a safe context, without putting the participant’s health at risk. As this research normally involves using treatments with no actual benefit, or even inducing false beliefs of effectiveness, it would be unethical to conduct such studies with real health outcomes. Additionally, it is sometimes possible to use samples of real patients who deal with fictitious or imagined health outcomes in the context of a causal learning experiment (Meulders et al., 2018), which helps to alleviate the limitations of ecological validity while using highly controlled procedures.

This line of research that uses causal learning experiments to study health beliefs has shown some promising advances. For example, it is possible to predict which conditions will make patients and users more vulnerable to pseudomedicine and bogus health claims (Blanco et al., 2014; Blanco and Matute, 2020), to discover situations in which previously acquired beliefs interfere with actual effectiveness (Yarritu et al., 2015), to investigate how health beliefs are affected by biases in Internet search (Moreno-Fernández and Matute, 2020), to explain why certain patients are hypersensitive to pain symptoms (Meulders et al., 2018), and to improve the effect of placebos (Yeung et al., 2014). This knowledge has the potential to offer a valuable foundation for designing interventions aimed at debiasing dysfunctional beliefs in real life settings (Lewandowsky et al., 2012; Macfarlane et al., 2020).

### Exploring Health Beliefs in the Laboratory

Most causal learning experiments exploit a basic principle of causality: causes and effects (outcomes) correlate with each other, unless a third factor masks this relationship. Since causality cannot be directly observed (Hume, 1748), people use this simple principle and rely on a proxy measure, the contingency between the cause and the outcome, to estimate causality (Allan, 1980; Wasserman et al., 1996; Vadillo et al., 2005; Blanco et al., 2010). In a simple situation with only one binary cause and one binary outcome, the contingency can be computed by means of the Δp index (Allan, 1980). This is simply the result of subtracting the probability of the outcome occurring given that the cause occurred, P(O| C), minus the probability of the outcome occurring given that the cause did not occur, P(O| ∼C). Large values of Δp correspond to situations in which the cause increases or decreases the probability of the outcome beyond the base-rate, P(O| ∼C). The larger this difference is, the stronger the association between cause and outcome, and therefore the higher the chances that there is a causal link. According to previous research, this is how probabilities could produce causal beliefs in many situations (Perales et al., 2016).

In the context of judging a treatment’s effectiveness, this reasoning amounts to computing how often the symptomatic episodes appear during the treatment, P(O| C), compared to how frequent they are without the treatment, P(O| ∼C). This comparison renders fairly in randomized controlled trials, in which two comparable groups of patients are recruited (i.e., experimental vs. control, or treatment vs. placebo). That is, clinicians often form their judgments on the effectiveness of a treatment after carefully comparing the two groups, and ensuring that occurrences of symptom remission are more frequent in the treatment group than they are in the control group. However, although this reasoning applies well to clinicians and researchers, patients often lack the resources to base their decisions on such complete information. Rather, they must form their beliefs of effectiveness on the basis of a more limited comparison: how often symptoms were observed before the treatment started vs. how often they occur during the treatment, on the same patient (usually, themselves). Most causal learning experiments do not take into account this limitation, and instead provide participants with information about a series of different patients (Blanco et al., 2014; Matute et al., 2019). This is useful to investigate the formation of causal knowledge in general, but it is not realistic when applied to the case of patients’ beliefs of effectiveness, as the procedure clearly departs from the actual experience of patients with their own treatments. In the current research, we propose a more natural setting to investigate the formation of beliefs of effectiveness, by presenting information of a single patient previous to, and during, a treatment (see a related approach in Blanco and Matute, 2020).

Previous experiments that used causal learning paradigms suggest that people can often be accurate in their judgments of causality (Shanks and Dickinson, 1987; Wasserman, 1990; Blanco et al., 2010), being generally sensitive to the actual contingency presented in the experiments. However, researchers have also reported systematic deviations, or biases. In particular, when the probability of the desired outcome is high, judgments tend to be higher even in null contingency conditions (Alloy and Abramson, 1979; Buehner et al., 2003; Blanco et al., 2014, 2020; Chow et al., 2019), contributing to what has been called a “causal illusion.” This is a bias consisting of the belief in a causal link that is actually inexistent (Matute et al., 2015; Matute et al., 2019). The causal illusion bias share some features with other phenomena like the classical illusory correlation effect (Chapman and Chapman, 1967, 1969), and pseudocontingencies (Kutzner et al., 2011; Fiedler et al., 2009).^1^ Despite their different explanations and assumptions, all these phenomena coincide in the importance of event probabilities, such as the probability of the cause and the probability of the outcome, when judging causal relationships.

Thus, the causal illusion (as well as the other related biases) has been suggested to underlie many beliefs related to treatment effectiveness, and in particular those concerning pseudomedicines. These are treatments claiming to be effective, despite the lack of scientific evidence supporting levels of effectiveness higher than those of placebo (Lilienfeld et al., 2014; Macfarlane et al., 2020). The rationale is that, when diseases have a high chance of spontaneous remission, people systematically overestimate the effectiveness of treatments, even of those treatments that are completely unable to produce an effect. This could have serious consequences in real-life, as patients may grant undeserved trust and reliability to treatments that produce no actual benefit, thus losing the therapeutic opportunity (Freckelton, 2012).

By contrast, little research has paid attention to another possibility: that patients may also underestimate the effectiveness of actually valid treatments. As we will show, we have reasons to expect that causal learning can also produce this underestimation effect under some circumstances (see an example in Yarritu et al., 2015). For instance, by virtue of the biasing effect of the probability of the remissions that we described above, a treatment might appear as not effective when used on a disease with frequent symptomatic episodes, compared to a mild disease with less frequent symptoms.

### Overview of the Experiments

In the current research, we use a causal learning procedure to experimentally study how people form beliefs of effectiveness for a fictitious treatment. Specifically, we present a medicine that is able to produce a moderate improvement in symptoms (i.e., a medicine with moderate contingency with symptom remission), and compare the perceived effectiveness in two situations: a disease with a high probability of symptomatic episodes, and a disease with a low probability of symptomatic episodes. Since the medicine equally works to reduce the frequency of episodes in both scenarios, one would expect similar ratings of effectiveness. However, the probability of the outcome (in this case, the observation of symptom remissions) could bias the judgments, producing the impression that the medicine is working better in the group in which symptoms had lower base-rates. In contrast with most previous studies on causal learning, we provide the information of the treatment effectiveness on a more natural fashion, which implies: (a) describing first how likely symptoms are before the treatment, and then how they respond to the introduction of the treatment, and (b) that the information given through a series of trials concerns only one patient, observed through time. This presentation format aims to mirror the chronology and generalization ability of the observations made by patients in real life.

## Ethics Statement

The procedure was revised and approved by the Ethical Review Board of the University of Deusto. The participants were informed before the experiment that they could quit the study at any moment by closing the browser window. No personal information (i.e., name, IP address, e-mail) was collected. We did not use cookies or other software to covertly obtain information from the participants. All measures, groups and conditions are disclosed. Data, materials, and R scripts for the three experiments are publicly available at the Open Science Framework: https://osf.io/emzbj/.

## Experiment 1

Experiment 1 uses a causal learning task to investigate the question of whether the effectiveness of a medicine can be underestimated if the disease has a high base-rate of symptomatic episodes. We expect that diseases that produce frequent observations of symptoms would create the impression that the treatment is not working as effectively as a treatment used for a disease with less frequent symptomatic episodes.

### Method

#### Participants

We initially planned a sample of 100 participants, which would allow for the detection of effects of *d* ≥ 0.57 in the difference between two groups at 80% power. However, data from one subject were not recorded due to technical errors. Thus, 99 Internet users (45 male, with age *M* = 31.38, SD = 9.88) participated anonymously through the Prolific Academic platform (Palan and Schitter, 2018), in exchange for money (0.80£ for about 10 min). The program randomly assigned 52 participants to the Infrequent group, and 47 to the Frequent group.

#### Procedure and Design

We adapted the standard trial-by-trial contingency learning task (Wasserman et al., 1990) that is extensively used to study human learning. The experiment was programmed in *JavaScript* to run online using a web browser. The instructions (available at the Open Science Framework, https://osf.io/emzbj/) asked participants to imagine that they were suffering from a fictitious disease called *Hamkaoman Syndrome*, which produces severe headaches. However, this symptom appears from time to time. Participants were told that the fictional drug *Batatrim* was a potential treatment for this disease if taken on a daily basis, but it may not work equally well for all people (i.e., “*Perhaps it works in your case, but we don’t know until we try*”). The goal of the task was to use the information to find out whether Batatrim works to stop the headaches.

**FIGURE 1:**
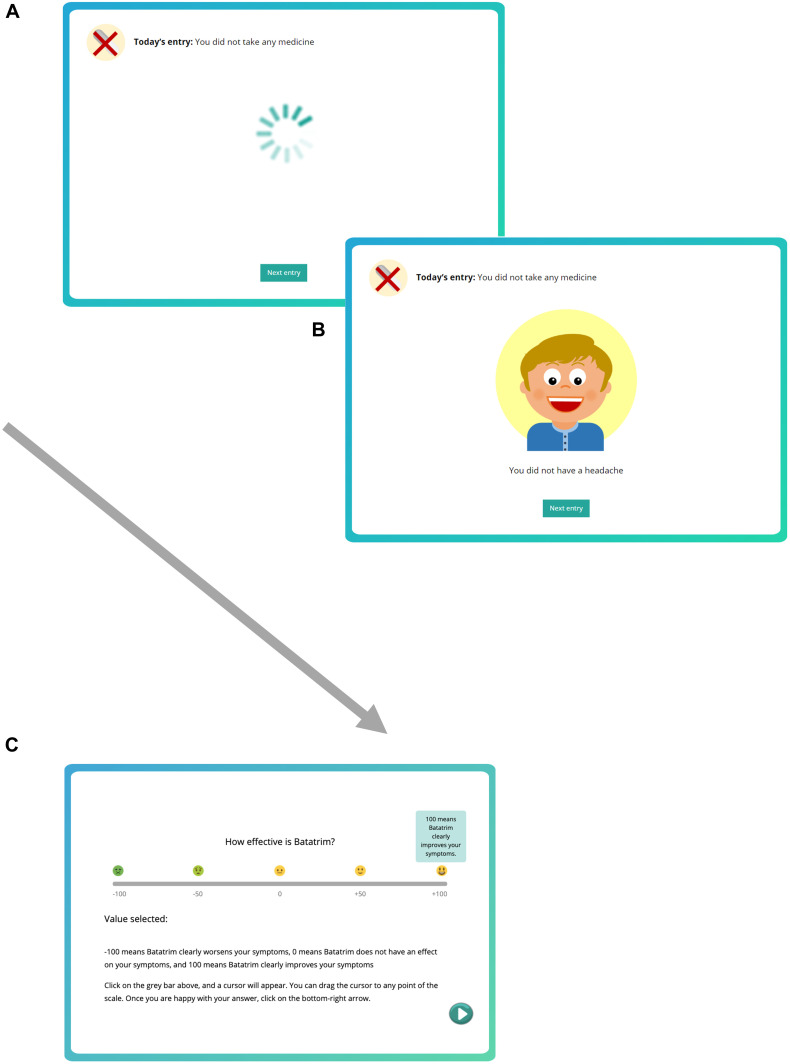
Screenshots showing the contingency learning task. **(A)** At the beginning of the trial, the information about the medicine (top part of the screen) is shown for 1 s. **(B)** Then, the information about the presence or absence of the symptoms is shown in the center of the screen (in this example, the patient did not report symptoms). Pressing the “Next entry” button leads to next trial after a delay (ITI) of 500 ms in which the screen is cleared. **(C)** After the training session, we collect an effectiveness judgment on a –100 to +100 scale.

Then, the training started by presenting a series of 40 records sequentially. Each record corresponded to one day, and displayed information about (a) whether the patient took Batatrim that day and, after a delay of 1 s, (b) whether the patient reported a headache (see Figures 1A,B). This information remained on the screen until the button “Next” was clicked, which proceeded to the next trial (after an inter-trial-interval of 500 ms).

The training comprised two consecutive phases. During Phase 1, as the instructions indicated, participants observed the records corresponding to the time before the treatment had started (“*In the first round of records, you will observe the diary entries corresponding to the time before you had any treatment, when you were just waiting for the doctor to give you Batatrim.*”). That is, Phase 1 contained 20 medicine-absent trials, in which either the patient reported a headache or not, and did not take any drug, therefore it conveyed the information to compute P(O| ∼C). Then, in Phase 2, participants started observing the 20 records that corresponded to the time after the treatment had started (“*You have already learned about the symptoms produced by the Hamkaoman Syndrome when no treatment is given. Now, your pills have arrived, and you will start taking Batatrim on a daily basis.*”). This means that only medicine-present trials were shown in Phase 2, which serves to compute P(O| C). The order of the trials within each phase (outcome-present or outcome-absent) was randomly determined for each participant.

Table 1 summarizes the experimental design. In the Frequent group, the symptoms were initially very frequent: 14/20 trials in Phase 1 (before treatment), and 8/20 in Phase 2 (during treatment). By contrast, in the Infrequent group, the symptoms were reported less often: 8/20 trials in Phase 1, and 2/20 in Phase 2. However, the objective contingency between treatment and symptom occurrence was the same in both groups. In the Frequent group, the contingency is computed as P(O| C) – P(O| ∼C) = 0.4–0.7 = −0.3; and in the Infrequent group it yields the same number, P(O| C) – P(O| ∼C) = 0.1–0.4 = −0.3. That is, according to the contingency rule for determining effectiveness (Δp), the two groups were depicting a medicine that was equally effective (a difference of 30% in the symptoms occurrence, in absolute terms), although they differed in the symptom base-rate.

**TABLE 1:** Design of Experiment 1.

**Group**	**Phase 1**	**Phase 2**	**P(O| ∼C)**	**P(O| C)**	**Contingency (Δp)**
Frequent	Symptoms reported: 14/20 trials	Symptoms reported: 8/20 trials	0.70	0.40	−0.30
Infrequent	Symptoms reported: 8/20 trials	Symptoms reported: 2/20 trials	0.40	0.10	−0.30

*The two groups differ in the base-rate with which the symptoms appeared. The medicine was equally effective in both groups according to the contingency rule Δp, because the difference in the symptom probability before and after treatment was the same in both groups, in absolute terms.*

After the sequence of 40 trials (20 in each phase), participants were asked several questions. First, we collected an effectiveness judgment (i.e., “*How effective is Batatrim?*”), which was our main dependent variable. The judgment was collected on a scale from −100 (“*Batatrim clearly worsens your symptoms*”) to 0 (“*Batatrim does not have an effect on your symptoms*”), to +100 (“*Batatrim clearly improves your symptoms*”). To help interpret the response scale, we included five evenly separated small pictures of faces ranging from −100 (sick face) to +100 (happy face). When participants hovered the mouse pointer over these pictures, a small box appeared with a verbal label as shown in Figure 1C. No time constraints were imposed to answer these questions.

Second, we asked two conditional probability questions (in random order for each participant): P(O| C) judgment (“*Imagine a different person who suffers from the same syndrome. This person takes Batatrim on 100 consecutive days. Out of these 100 days in which the person takes Batatrim, on how many of them will the person report having headaches?*”), and P(O| ∼C) judgment (“*Imagine a different person who suffers from the same syndrome. This person does not take Batatrim on 100 consecutive days. Out of these 100 days in which the person does not take Batatrim, on how many of them will the person report having headaches?*”). These two pieces of information, combined, serve to compute the contingency between treatment and symptoms, and hence are necessary to correctly assess effectiveness. By examining these two questions, we will be able to detect whether participants correctly encode the two probabilities.

Finally, we requested a judgment about the tendency to opt for an alternative treatment different from Batatrim (“*If you had the chance, would you stick to your current treatment with Batatrim, or would you try a different treatment?*”). This was answered on a scale with five options (“*I’m sure I would stick to Batatrim*” / “*I would probably stick to Batatrim*” / “*I don’t know*” / “*I would probably try a different treatment*” / “*I’m sure I would try a different treatment*”). We expected that participants who felt that the medicine was not working well would be more likely to stop taking it and try a different treatment.

### Results and Discussion

**FIGURE 2:**
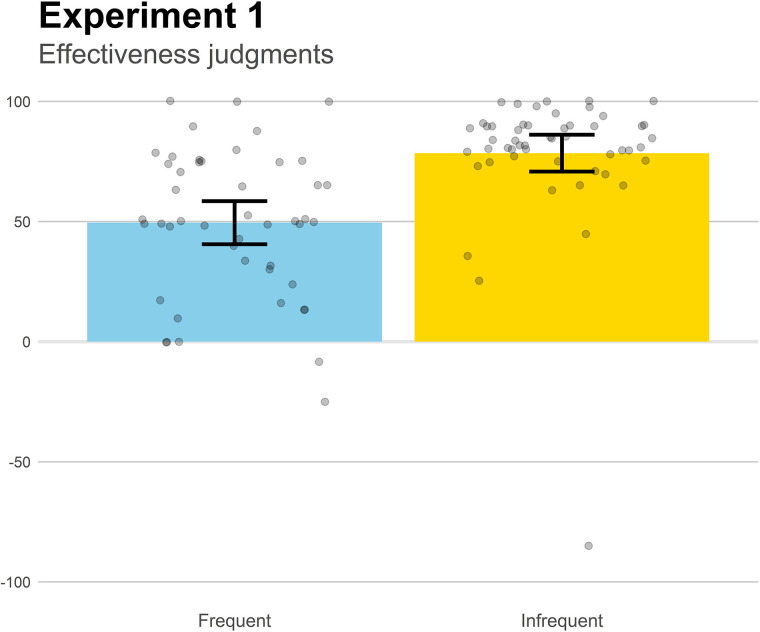
Mean effectiveness judgments in Experiment 1. Higher positive values indicate a strong belief that the medicine works to reduce the symptoms. Jittered data points are superimposed to the plot (to avoid overplotting, the placement of data points of a given condition along the *x*-axes is random). Error bars depict 95% confidence intervals for the mean.

The main results are those obtained from the effectiveness judgments, depicted in Figure 2. Although the medicine was identically effective in both groups according to the contingency information, the effectiveness judgments were significantly higher in the Infrequent group (which featured a lower symptom rate before the medicine was taken) than in the Frequent group, *t*(97) = 4.96, *p* < 0.001, *d* = 0.998. This suggests that those diseases that course with frequent symptomatic episodes will produce an underestimation of the actual effectiveness of the treatment relative to those with less frequent symptoms.

Next, we examine the judgments measuring the tendency to switch to alternative treatments, whose descriptive statistics appear in Table 2. The judgments could range between 1 (“*I’m sure I would stick to Batatrim*”) and 5 (“*I’m sure I would try a different treatment*”). These judgments were significantly higher in the Frequent group than in the Infrequent group, *t*(97) = 4.22, *p* < 0.001, *d* = 0.850. That is, those participants who observed a disease with frequent symptomatic episodes were not only more likely to produce lower estimates for the effectiveness of the medicine, but they were additionally less willing to adhere to the treatment with Batatrim, despite the medicine being identically effective in the two groups.

**TABLE 2:** Descriptive statistics for the alternative treatment judgments in the three experiments.

**Experiment**	**Group**	**Mean**	**SD**
Experiment 1	Frequent	2.85	1.23
	Infrequent	1.90	1.00
Experiment 2	Frequent-Experimental	2.66	1.18
	Infrequent-Experimental	1.73	0.83
	Frequent-Control	3.96	0.99
	Infrequent-Control	3.87	0.95
Experiment 3	High Continency-Large Change	1.98	0.91
	Low Continency-Large Change	1.91	0.85
	Low Continency-Small Change	3.03	1.07

*The judgment was collected on a scale from 1 to 5 (1: “I’m sure I would stick to Batatrim”; 2: “I would probably stick to Batatrim”; 3: “I don’t know”; 4: “I would probably try a different treatment”; 5: “I’m sure I would try a different treatment”).*

**FIGURE 3:**
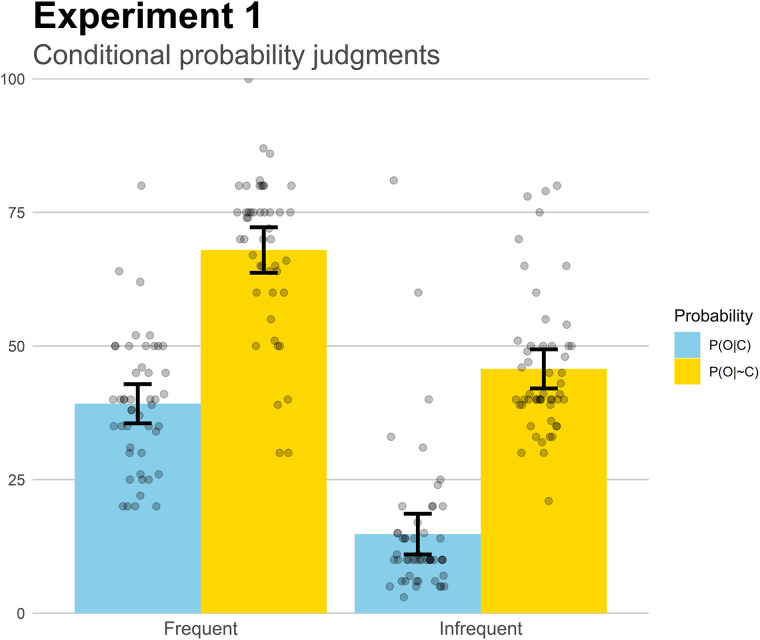
Mean conditional probability judgments in Experiment 1. Jittered data points are superimposed to the plot (to avoid overplotting, the placement of data points of a given condition along the *x*-axes is random). Error bars depict 95% confidence intervals for the mean.

Finally, we analyzed the conditional probability judgments to gain insight into how participants learned these two pieces of information, the probability of symptoms when the medicine was taken, P(O| C) and the probability of symptoms when no medicine was taken, P(O| ∼C). These judgments are depicted in Figure 3. We conducted a mixed 2 (Group) × 2 (Probability), revealing a main effect of Group, *F*(1,97) = 117.0, *p* < 0.001, $\eta_{p}^{2}=0.55$. Overall, probability judgments were greater in the Frequent group than in the Infrequent group, which is consistent with the actual symptom probabilities in each group. We also found a main effect of Probability, *F*(1,97) = 327.91, *p* < 0.001, $\eta_{p}^{2}=0.77$, which just reflects the fact that the symptoms reduced their frequency from Phase 1 to Phase 2 (i.e., the medicine was effective). Importantly, there was no interaction, *F* < 1. To better interpret these results (and those of subsequent experiments, with additional groups), we computed a “perceived contingency score” by subtracting the two conditional probability judgments following the Δp rule, i.e., P(O| C)-P(O| ∼C). These scores can then be interpreted as the amount of contingency that a participant perceived, based on the conditional probability ratings. The resulting values showed no differences between groups, *t*(97) = 0.65, *p* = 0.51, *d* = 0.13, indicating that the perceived contingency was the same in both base-rate groups, as the conditional probability estimations only differed between groups in their absolute values. Taken together, the results suggest that participants were able to capture accurately the probabilities involved in the computation of contingency, as the mean estimations were close to the actual values presented in the task. Therefore, the underestimation of effectiveness that we reported above cannot be explained as a failure to learn the conditional probabilities.

## Experiment 2

Experiment 1 successfully showed that the base-rate of the symptomatic episodes can bias the judgments of treatment effectiveness: diseases with a higher probability of symptoms produced lower perceived effectiveness, even if the actual contingency was identical. This aligns with the evidence obtained in different situations (e.g., null contingencies), and also with results from experiments conducted in related paradigms (e.g., pseudocontingencies, Kutzner et al., 2011).

Still, our results could be interpreted as if our participants were simply ignoring the contingency information, guiding their judgments by the probability of symptoms only. That is, it could be possible that if a medicine drives the probability of symptoms close to zero, it would be judged as effective even if the initial base-rate without treatment was also small, as people could just ignore the initial base-rate. In fact, as we mentioned above, there is ample empirical evidence indicating that judgments of causality can be strongly biased by the probability of the outcome, at least in null contingency situations (Alloy and Abramson, 1979; Buehner et al., 2003; Blanco et al., 2014; Chow et al., 2019; Blanco and Matute, 2020).

Experiment 2 aims to replicate the findings of Experiment 1, while introducing two control groups in which the actual contingency between the treatment and symptom remissions is zero: In these two control groups, the probability of the symptoms is the same before and after the treatment (i.e., the medicine does not work at all). These two probabilities match those of the two experimental groups when taking the medicine, P(O| C), which are identical to those used in Experiment 1. That is, for half of the participants, symptoms will be frequent, and for the other half they will be infrequent. Orthogonally, for half of the participants, the medicine will work (by reducing the symptom probability in 30%, in absolute terms), whereas for the other half it will not work at all. Thus, if participants judge the effectiveness of the treatment only by attending to the frequency of the symptoms and ignoring the contingency, then the control groups would not differ from the experimental groups, revealing that participants are only biased by the base-rate of the effect. Conversely, if participants do take into account contingency, they should note that control medicines are not effective.

### Method

#### Participants

The planned sample size was *N* = 200, which allows detecting effects of *d* ≥ 0.57 at 80% power. Data from three participants were not recorded due to technical errors. The final sample consisted of 197 anonymous Internet users (105 male, 91 female, 1 non-binary, with age *M* = 30.8, SD = 11.3), who participated through Prolific Academic (Palan and Schitter, 2018) in exchange for money (0.80*textsterling* for about 10 min). The program randomly assigned 52 to the Frequent-Control group, 47 to the Frequent-Experimental group, 47 to the Infrequent-Control group, and 51 to the Infrequent-Experimental group.

#### Procedure and Design

The procedure was identical to that in Experiment 1. The only change was the inclusion of two new groups that work as control conditions (see the design in Table 3). In these groups, the actual contingency between medicine and recovery from the symptoms was null, which means that the medicine was completely ineffective. That is, in addition to the two groups already present in Experiment 1, we had the Infrequent-Control group, which showed a base-rate of symptomatic episodes of 0.10 (i.e., 2/20 trials), both in Phase 1 and in Phase 2; and the Frequent-Control group, which showed a base-rate of symptomatic episodes of 0.40 (i.e., 8/20 trials), both in Phase 1 and in Phase 2. In sum, now we have included null-contingency controls for the two base-rate conditions that were previously tested. This will allow us to compare the two factors: will judgments depend on the symptoms base-rate, or on contingency (or both)?

**TABLE 3:** Design of Experiment 2.

**Group**	**Phase 1**	**Phase 2**	**P(O| ∼C)**	**P(O| C)**	**Contingency (Δp)**
Frequent-Experimental	Symptoms reported: 14/20 trials	Symptoms reported: 8/20 trials	0.70	0.40	–0.30
Infrequent-Experimental	Symptoms reported: 8/20 trials	Symptoms reported: 2/20 trials	0.40	0.10	–0.30
Frequent-Control	Symptoms reported: 8/20 trials	Symptoms reported: 8/20 trials	0.40	0.40	0.00
Infrequent-Control	Symptoms reported: 2/20 trials	Symptoms reported: 2/20 trials	0.10	0.10	0.00

*In addition to the two experimental groups, identical to those in Experiment 1, this experiment included two control groups in which the probability of the symptoms during the treatment were the same as in the experimental groups, but the contingency was null (the medicine did not work at all).*

### Results and Discussion

**FIGURE 4:**
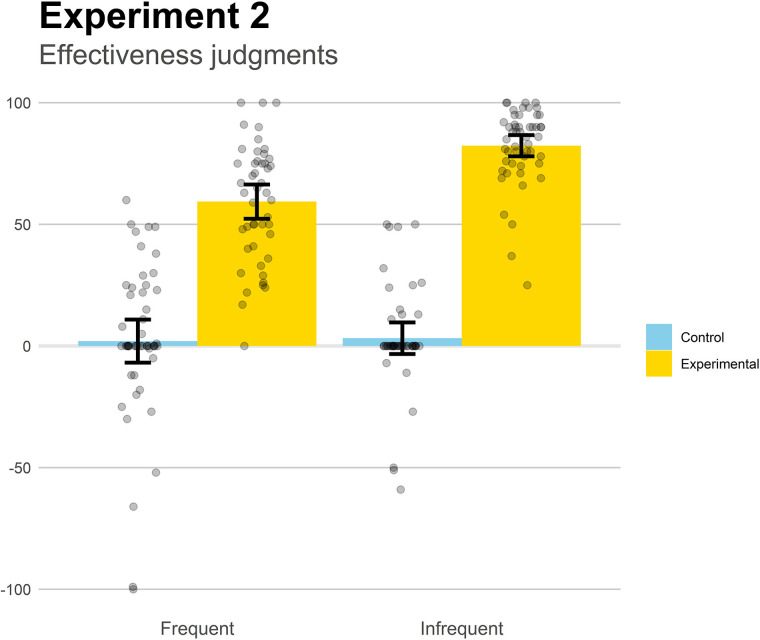
Mean effectiveness judgments in Experiment 2. Higher positive values indicate a strong belief that the medicine works to reduce the symptoms. Jittered data points are superimposed to the plot (to avoid overplotting, the placement of data points of a given condition along the *x*-axes is random). Error bars depict 95% confidence intervals for the mean.

The mean effectiveness judgments are displayed in Figure 4. They were submitted to a 2 (Base-rate) × 2 (Contingency) factorial ANOVA. The main effect of Contingency was significant, *F*(1,193) = 392.4, *p* < 0.001, $\eta_{p}^{2}=0.67$, indicating that participants were sensitive to contingency, producing higher judgments when the medicine was effective (Experimental groups) than when it was not effective (Control groups). The main effect of base-rate was also significant, *F*(1,193) = 12.3, *p* < 0.001, $\eta_{p}^{2}=0.06$, meaning that the infrequent groups produced stronger beliefs of effectiveness. Finally, the interaction, *F*(1,193) = 10.0, *p* = 0.002, $\eta_{p}^{2}=0.05$, indicated that, while the two experimental groups were sensitive to base-rate, meaning that we successfully replicated the effect reported in Experiment 1, *t*(96) = 5.67, *p* < 0.001, *d* = 1.15, the two control groups did not differ from each other, *p* = 0.827. That is, base-rate information only affected the effectiveness judgments in the two contingent groups.

The judgments about the likelihood to switch to an alternative treatment (Table 2) aligned with the previous conclusions. They showed, again, the main effect of Contingency, *F*(1,193) = 148.55, *p* < 0.001, $\eta_{p}^{2}=0.43$, the main effect of base-rate, *F*(1,193) = 13.08, *p* < 0.001, $\eta_{p}^{2}=0.06$, and the interaction, *F*(1,193) = 8.92, *p* = 0.003, $\eta_{p}^{2}=0.04$. The two experimental groups differed from each other as in Experiment 1, *t*(96) = 4.56, *p* < 0.001, *d* = 0.92, thus replicating the previous result, while the two controls did not differ, *p* = 0.648. In sum, the results concerning the alternative treatment judgments were consistent with those of the effectiveness judgments: participants in the control groups were more likely to try a different therapeutic option, while in the experimental group the symptom base-rate mattered, so that the higher the symptom base-rate, the more unlikely they were to adhere to the treatment.

**FIGURE 5:**
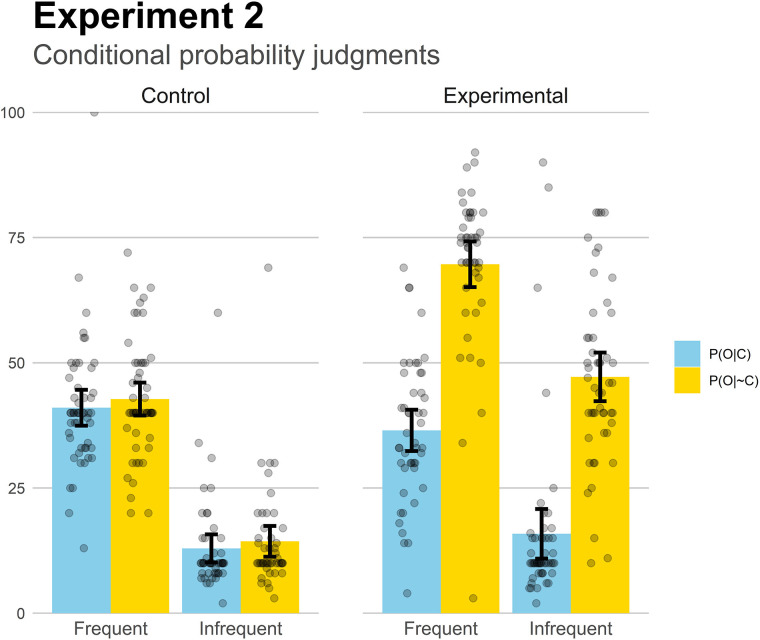
Mean conditional probability judgments in Experiment 2. Jittered data points are superimposed to the plot (to avoid overplotting, the placement of data points of a given condition along the *x*-axes is random). Error bars depict 95% confidence intervals for the mean.

Finally, we analyzed the conditional probability judgments (Figure 5). The two Experimental groups replicated the results from Experiment 1: P(O| C) was estimated higher than P(O| ∼C) in both base-rate levels, *t*(46) = 11.0, *p* < 0.001, *d* = 1.60 (Frequent), and *t*(50) = 10.6, *p* < 0.001, *d* = 1.49 (Infrequent), while overall both probabilities were close to the actual values. In the control groups, there were no differences between the two conditional probabilities, which is consistent with the low effectiveness judgments, *p* = 0.41 (Frequent), and *p* = 0.29 (Infrequent). Like in Experiment 1, to make the interpretation of these results easier, we decided to compute a “perceived contingency” score by subtracting the judgments to the P(O| C) and to the P(O| ∼C) questions, thus following the contingency equation Δp. A 2 (Base-rate) × 2 (Contingency) ANOVA on these perceived contingency values revealed a main effect of Contingency, *F*(1,193) = 156.89, *p* < 0.001, $\eta_{p}^{2}=0.45$, with no other significant effects or interaction (both *F*s < 0.2). The effect of contingency means that the two experimental groups (who were exposed to a positive contingency) perceived higher contingency levels than did the two control groups (who were exposed to a null contingency), irrespective of the differences in base-rate. Thus, the experimental groups replicated the results of Experiment 1, by not finding an effect of base-rate on the perceived contingency: it seems that the perceived contingency was the same regardless of the frequency of presentation of the symptoms.

## Experiment 3

The results of Experiment 2 suggested that the effectiveness judgments produced by participants were affected by the symptom base-rate. However, participants were not completely ignoring the contingency information, as they, at least, were able to discriminate between a low/moderate contingency level (0.30) and a null contingency (0). The question is: how do participants use base-rate information to form their judgment?

Contingency, as described in section “Introduction,” is an objective rule used to assess treatment effectiveness, which in principle allows the comparison of treatments for different cases, with different levels of symptom frequency. The two previous experiments suggested that participants, however, produce effectiveness judgments that are not only determined by contingency, but also biased by the frequency of the symptoms. It is possible to further investigate the way in which people use symptom base-rates when judging effectiveness. In fact, in our previous experiments, we fixed the contingency level to a given value of 0.30 (or zero in the control groups in Experiment 2), which means that the treatment always produced the same amount of change in the symptom probability *in absolute terms*. However, the groups differed in the amount of change in the symptom probability *relative to the base-rate level*. That is, when the treatment reduces the symptom occurrence from 0.70 to 0.40 (i.e., group Frequent), the absolute difference, or contingency, is 0.30, but the amount of reduction relative to the base-rate is 43%, i.e., (0.40–0.70)/0.70 = 0.43. By contrast, when the treatment reduces the symptom occurrence from 0.40 to 0.10 (i.e., group Infrequent), the absolute change remains 0.30 but the relative change is larger, 75%, i.e., (0.10–0.40)/0.40 = 0.75. Thus, it is possible that participants in our previous experiments were judging effectiveness by using the change in the symptoms proportional to the base-rate, rather than by using the absolute difference (contingency). This would be a different strategy to deal with effectiveness information that takes into account base-rates, and that could explain our results so far (note that using this strategy can also explain the results from the two groups with a null contingency).

Therefore, we designed Experiment 3 to test for this possibility. In Experiment 3, the groups differed either in the contingency level (high vs. low) or in the amount of change proportional to the base-rate (small vs. large). The parameter constellations were chosen such that the two possible drivers for participants’ judgments (absolute differences vs. relative differences) could be pit against each other.

### Method

#### Participants

We planned a sample size of *N* = 150 for this design of three groups (50 participants per group), which allows detecting effects for the difference between pairs of groups of *d* ≥ 0.57 at 80% power. Data from one participant were lost due to technical errors/connection issues. The final sample consisted of 149 participants (70 women, 79 men, with age *M* = 27.3, SD = 8.66), recruited in the same way as in the previous experiments. The program randomly assigned 55 to the High Contingency-Large Change group, 57 to the Low Contingency-Large Change group, and 37 to the Low Contingency-Small Change group.

#### Procedure and Design

The procedure was identical to the previously reported experiments, except for the probability of observing symptoms during the training, which was manipulated across the three groups to obtain two different levels of contingency and two different levels of the change proportional to the base-rate (Table 4). That is, in the High Contingency-Large Change group, the contingency between the treatment and the symptom occurrence was high (−0.60) in absolute terms, and the change proportional to the symptom base-rate was large (a reduction of 75% from the initial symptom base-rate); in the Low Contingency-Large Change group, the contingency was low (−0.30), but when considered as a proportion of the initial symptom base-rate, the change was still large (a reduction of 75% of the initial symptoms); finally, in the Low Contingency-Small Change group, the contingency was low (−0.30), and the change proportional to the base-rate was small (a reduction of 37.5% of the initial symptoms). By comparing these groups pairwise, as they share one of the parameters (either contingency or proportional change) but not the other, we can eventually find out which of the two parameters more clearly affects judgments of effectiveness.

**TABLE 4:** Design of Experiment 3.

**Group**	**Phase 1**	**Phase 2**	**P(O| ∼C)**	**P(O| C)**	**Contingency (Δp)**	**Change (%)**
High Contingency-Large Change	Symptoms reported: 16/20 trials	Symptoms reported: 4/20 trials	0.80	0.20	−0.60	75
Low Contingency-Large Change	Symptoms reported: 8/20 trials	Symptoms reported: 2/20 trials	0.40	0.10	−0.30	75
Low Contingency-Small Change	Symptoms reported: 16/20 trials	Symptoms reported: 10/20 trials	0.80	0.50	−0.30	37.5

*In this experiment, the probability of observing a symptom was manipulated between groups so that two of them showed the same low level of contingency (groups Low Contingency-Large Change and Low Contingency-Small Change), contrasting with a high contingency group (High Contingency-Large Change). Additionally, the amount of change between phases proportional to the symptom base-rate was identical (large) in two groups (High Contingency-Large Change and Low Contingency-Large Change), despite they diverged in their contingency level, and different from the Low Contingency-Small Change group.*

### Results and Discussion

**FIGURE 6:**
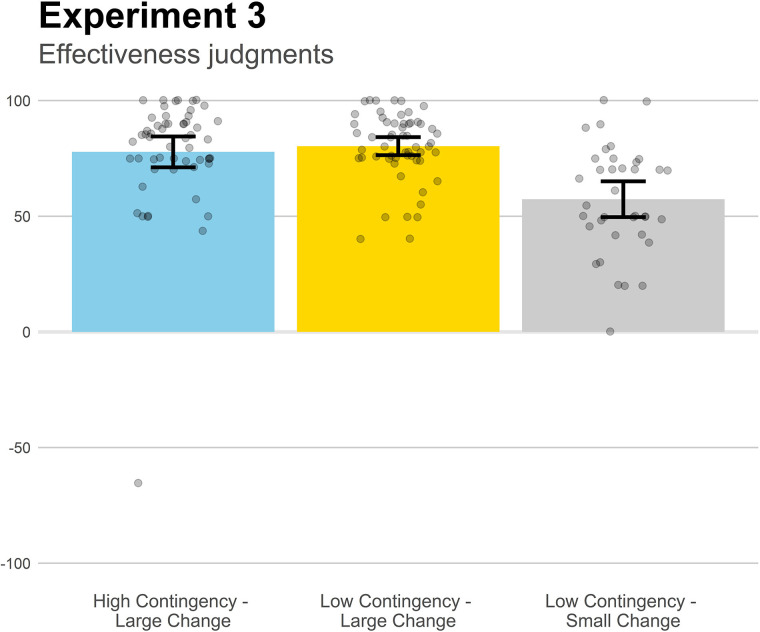
Mean effectiveness judgments in Experiment 3. Higher positive values indicate a strong belief that the medicine works to reduce the symptoms. Jittered data points are superimposed to the plot (to avoid overplotting, the placement of data points of a given condition along the *x*-axes is random). Error bars depict 95% confidence intervals for the mean.

Figure 6 contains the mean effectiveness judgments in the three groups of Experiment 3. We were only interested in the comparisons between the groups that shared one parameter value (either contingency or proportional change) and differed on the other. The Low Contingency-Large Change and the Low Contingency-Small Change groups, despite having identical contingency, differed significantly, *t*(92) = 5.87, *p* < 0.001, *d* = 1.24, suggesting that contingency was not a key aspect for effectiveness judgments, and rendering plausible that the proportional change played a role in this effectiveness assessment. This possibility was further reinforced by the finding that the Low Contingency-Large Change and High Contingency-Large Change groups, which shared the same proportional change but show different contingency, did not significantly differ from each other, *p* = 0.81.

The judgments about the likelihood to switch to an alternative treatment (Table 2) showed the same pattern as the effectiveness judgments: In the Low Contingency-Large Change group, participants were significantly more likely to stick to the treatment than they were in the Low Contingency-Small Change group, *t*(92) = 5.61, *p* > 0.001, *d* = 1.18. As it happened with effectiveness judgments, no differences were found in the likelihood to adhere to the actual treatment when comparing groups with similar proportional change, i.e., Low Contingency-Small Change vs. High-Small, *p* = 0.92.

**FIGURE 7:**
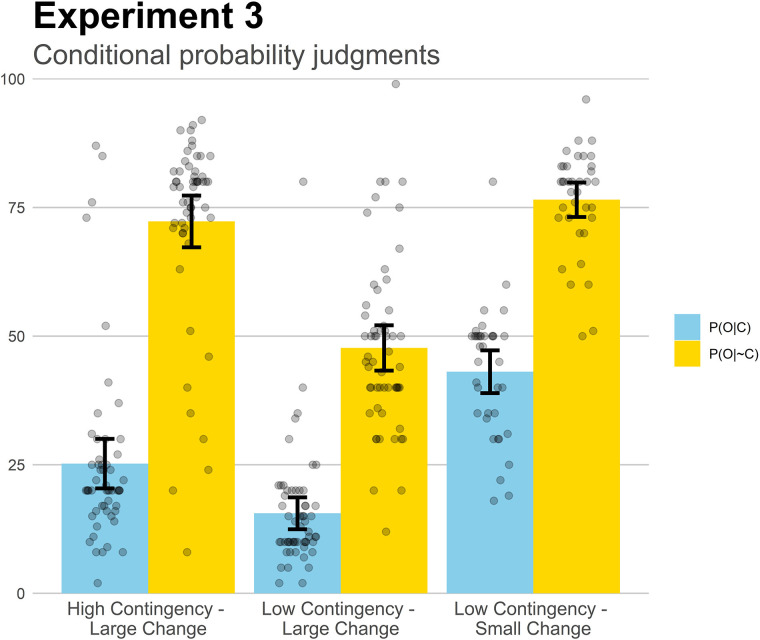
Mean conditional probability judgments in Experiment 3. Jittered data points are superimposed to the plot (to avoid overplotting, the placement of data points of a given condition along the *x*-axes is random). Error bars depict 95% confidence intervals for the mean.

Finally, Figure 7 depicts the conditional probability judgments for Experiment 3. Once again, the judgments were close to the actual values presented in the training. In all three groups, the difference between P(O| C) and P(O| ∼C) was significant (all *p*s < 0.001), consistent with the perception of at least some degree of effectiveness. Additionally, we used these conditional probability judgments to reconstruct the perceived contingency (by subtracting the two conditional probabilities) and the perceived proportional change between phases (by computing the contingency and dividing it by the symptom base-rate before the treatment). We found that groups with different contingency levels showed different perceived contingency scores: the High Contingency-Large Change group produced a larger difference between the conditional probabilities than did the other two groups, both *p*s < 0.007. On the other hand, groups with an identical contingency level did not differ in this measure: Low Contingency-Large Change vs. Low Contingency-Small Change, *p* = 0.95. Concerning the perceived proportional change, this score was higher for the groups with larger changes, even if they implied the same contingency: Low Contingency-Small Change differed both from High Contingency-Large Change and from Low Contingency-Large Change (both *p*s < 0.030). By contrast, groups with similar proportional change did not differ in this measure: Low Contingency-Large Change vs. High Contingency-Large Change, *p* = 0.998.

In a nutshell, it seems that effectiveness judgments were sensitive to proportional change in the conditional probabilities, but not to their absolute differences. This effect was also found in the desire to replace the treatment by an alternative. However, conditional probabilities seemed to be accurately captured.

## General Discussion

### Which Is the Rule for Estimating Effectiveness?

Beliefs of treatment effectiveness can be understood as the result of causal learning (Rottman et al., 2017), under the assumption that an effective treatment produces a change in the likelihood of symptom improvement compared to a control condition (e.g., taking no treatment). This allows us to investigate effectiveness beliefs by means of causal learning experiments, and to advance predictions based on the results described in this literature. Previous studies have focused on how completely ineffective medicines (e.g., pseudomedicines) can appear to be effective under some circumstances (Blanco et al., 2014; Matute et al., 2019). However, fewer experiments have been conducted to explore the possibility that actually effective treatments are seen as less effective due to the biases described in the causal learning literature.

Here, we have reported how beliefs of effectiveness are sensitive to the base-rate of the symptomatic episodes in a way that does not conform to the rule for computing contingency, Δp. That is, in Experiments 1 and 2, a fictitious medicine with a low/moderated contingency with health improvement (reduction of 0.30 in the probability of symptoms, in absolute terms) was tested in two different scenarios: a disease with high base-rate of symptoms and a disease with low base-rate of symptoms. Our results indicated that base-rates affected the judgments of effectiveness, so that a valid medicine was judged as less effective when the symptoms were very frequent before the treatment. This would modulate the perceived effectiveness of a treatment as a function of the symptom frequency, which could lead to mistaken conclusions when patients examine their treatments’ effectiveness, or when they compare between diseases or patients with diverging symptom base-rates. In fact, according to our results, it is those patients who show symptoms with greater probability who will be more likely to underestimate the effectiveness of a moderately valid treatment. The implication of this is that these patients who suffer from frequent symptomatic episodes should be carefully supervised, as we know that treatment effectiveness beliefs are core to treatment adherence (Leventhal et al., 1992; Dilla et al., 2009). Additionally, those patients who underestimate the effectiveness of their treatment will be probably at risk of replacing their scientifically valid treatment by a different, probably less effective one, or even by a pseudomedicine, as our experiments also reveal through the alternative treatment question. Not surprisingly, lack of trust in scientific medicine is one of the predictors of pseudomedicine usage (Macfarlane et al., 2020).

The underestimation of the effectiveness when the symptom base-rate is high (Experiment 1) could be due to participants judging effectiveness on the basis of how infrequent the symptoms are when the treatment is taken. That is, any medicine that drives the probability of symptoms close to zero (i.e., complete healing) would be judged as effective, while the initial base-rate without treatment could be ignored. This possibility was examined in Experiment 2, which included control groups with null contingency: that is, the symptom-base-rate was kept identical before and during the treatment. Since participants in Experiment 2 were able to discriminate between the two contingency levels while still replicating the bias reported in Experiment 1, it seems that people’s judgments are not entirely driven by the symptom level obtained at the end of the treatment.

Finally, Experiment 3 tested a potential way in which people could be using the symptom base-rate information when making their judgment, which is different from contingency. As we have described it, contingency is simply the difference between the symptom probability before and during the treatment, in absolute terms. Thus, it is an objective measure that is independent of the initial base-rate level. That is, a reduction of symptom probability from 0.70 to 0.40 is the same as one from 0.40 to 0.10. In this type of scenario, a contingency index, Δp (Allan, 1980), has been used as the traditional benchmark to assess causality and, hence, treatment effectiveness. However, people could be focusing on the reduction in symptom probability relative to the initial base-rate value, instead of in absolute terms. That is, when symptoms decrease from 0.40 to 0.10, they are reducing in 75% of the initial value. Experiment 3 presented three groups varying in either their contingency or their change proportional to the base-rate. Judgments were systematically guided by the change proportional to base-rate, rather than by contingency, suggesting that this is the way people use base-rate information to estimate effectiveness in this type of experiments. The explanation is compatible with all the results that we report in this article.

Is it reasonable to use proportional change, rather than absolute change (contingency) when assessing treatment effectiveness? In fact, researchers commonly use proportional change as a success index when testing the effectiveness of an intervention (especially in repeated-measures designs). For example, a treatment for depression could be regarded as useful if it reduces depressive symptoms by 10% from the baseline (see an example of the use of percent change from baseline, Lin et al., 2013). This is the logic underlying likelihood ratios (e.g., probability of the outcome given the treatment, relative to a control condition with no or other treatment) and odd ratios, which are common to estimate treatment effectiveness, test sensitivity, and risk in scientific studies (the same rationale is also present in the widely used Bayes Factors, Kass and Raftery (1995), which represent the support for one hypothesis relative to the null by means of an all-purpose likelihood ratio, although their computation is completely different). However, when used directly to assess the effectiveness of a treatment from the observation of the conditional probabilities, this approach can be problematic, and methodologists recommend to avoid it in most cases (Vickers, 2001; Tu, 2016). First, proportional change makes sense only with variables measured in ratio scales, in which zero is a meaningful value (fortunately, this condition holds in our case, as we are comparing probabilities). Additionally, note that, while contingency is an effectiveness measure that is insensitive to the symptom base-rate, the proportional change will strongly depend on this piece of information, so that those patients or conditions in which symptoms appear very often (i.e., high base-rate) will produce systematically smaller proportional changes than those in which symptoms are less frequent. Indeed, research works using this proportional change as outcome variable usually report strong correlations between the effectiveness of the manipulation and the baseline level (Tu and Gilthorpe, 2007; Tu, 2016), so that higher baseline levels apparently “reduce” the effectiveness. Moreover, despite it appearing to be an intuitive concept, presenting the information as proportional change can be confusing for patients. For example, when laypeople are presented with the results of a study on risk factors in terms of proportional change from baseline, they tend to erroneously interpret it as change in absolute terms (e.g., a reduction of 10% is interpreted as if a baseline score of 50 were reduced to 40, rather than 45) (Bodemer et al., 2014). Admittedly, there are situations in which proportional change could be a more useful measure of effectiveness than is direct difference (e.g., causes that produce a multiplicative effect), but most of times changes expressed as proportions are hard to generalize, as they depend on baseline levels that can vary between conditions or individuals (e.g., a change of 0.3 points in absolute terms can be small when the baseline is 0.9, but large when the baseline is 0.35). Thus, a direct difference measure such as the Δp index could be more versatile than likelihood ratios and related measures based on proportional change. In sum, proportional change from baseline is neither an accurate index for assessing treatment effectiveness, nor a good way to communicate it, at least in most situations. Hence, using proportional change could be considered a strategy that measures effectiveness, but in a suboptimal way that could lead to erroneous conclusions in some circumstances.

However, the finding that people spontaneously tend to use proportional change as an effectiveness index (as the results of Experiment 3 indicate) is interesting for theoretical reasons. Research on human causal and contingency learning has traditionally focused on objective measures such as Δp or similar rules (Perales and Shanks, 2007), not considering the possibility that participants use proportional change as a direct cue to causality assessment. Nonetheless, certain Bayesian theories of causal induction such as Causal Support (Griffiths and Tenenbaum, 2005) formalize causal inference in a way that involves likelihood ratios, that is, the probability of observing the data given one hypothesis (and model) relative to the probability of observing the data given an alternative one, which is structurally similar to a Bayes Factor (Kass and Raftery, 1995). For example, Causal Support computes a ratio of the likelihood of observing the current data under the model that assumes a causal link between cause and outcome, relative to the model that assumes no causal link, P(data| hypothesis, model_1_)/P(data| hypothesis, model_0_). The computation of Causal Support is more complex than merely comparing the two conditional probabilities, and it involves additional assumptions about causality. However, we mention it here because there could be some structural resemblance between the way the model computes causal strength (and the way Bayes Factors express support for a hypothesis) and the strategy apparently exhibited by our participants. Our experiments were not designed to investigate these questions, but the findings of Experiment 3 could inspire further studies to better understand how people incorporate base-rates to assess effectiveness and causality.

In fact, there is evidence that people use proportional change as a cue in completely different paradigms. For example, when they compare two numbers, people’s responses are affected by the ratio between the two quantities (i.e., “numerical size effect”; Moyer and Landauer, 1967). Additionally, studies on Bayesian reasoning also show that participants can use the information expressed as likelihood ratios to elaborate their judgments [although these judgments are often incorrect, especially when the information is given in terms of probabilities rather than natural frequencies (Gigerenzer and Hoffrage, 1995; Hoffrage et al., 2015)]. Nevertheless, this paradigm is quite different from ours: Bayesian reasoning tasks first provide the conditional probabilities and base-rates, and then ask about the probability that an individual observation corresponds to a given category (which requires using the base-rate information), whereas our contingency learning task provides a sample of observations already classified, and then requests a generalized rule (i.e., whether there is a causal link or not) that in principle should hold regardless of the particular base-rate observed. Further studies should examine the potential similarities and connections between these numerical cognition effects and contingency learning phenomena.

It is also worth discussing the results concerning the conditional probability judgments. Across the three experiments, we found that the departure from contingency was detected in effectiveness judgments, formulated as a causal question, but not in the conditional probability judgments. This is in line with recent studies on the causal illusion (Chow et al., 2019) and coincides with previous claims that, generally, causal estimations are more prone to bias than are other types of judgments, such as predictions (Vadillo et al., 2005). This also has theoretical implications: some authors have proposed that biases in causal learning are the result of processes that appear in the moment of emitting the judgment, rather than in the encoding phase (Allan et al., 2008). Indeed, in our experiments, the basic pieces of information needed to compute the contingency index Δp, P(O| C) and P(O| ∼C), seem to have been correctly acquired. Therefore, the effects we have described in this article might be explained by the strategies or rules that people use to combine the information and form their judgment (e.g., using proportional change instead of contingency), rather than by learning or encoding phenomena. However, we must remain cautious when interpreting the conditional probability judgments, as they were always requested after the effectiveness judgment, and therefore they could be contaminated.

### Methodological Aspects

Additionally, these experiments included several procedural and methodological innovations that depart from most previous literature, and that deserve discussion. First, most experiments using causal learning tasks in medical scenarios present the four types of trial (i.e., medicine-healing, medicine-no healing, no medicine-healing, and no medicine-no healing) in intermixed, often random, orders. Additionally, the information given on each trial concerns usually a different patient. Thus, the traditional task resembles a clinical study in which a sample of patients is examined, in no particular order. This causal learning task has advantages. For example, it prevents participants from assuming that trials are autocorrelated (i.e., that there is dependency between trials, so that the outcome of one trial can be affected by previous trials) and avoids order effects by randomizing the trial order. However, this procedure does not capture well the experience of patients who judge their own treatments, which is a highly common situation in real life. Patients cannot normally access a sample of participants to test the treatment. Rather, they can only test the effectiveness on themselves, and the information is, most of the time, examined in a particular order: first, they know how often the symptoms appear before the treatment, i.e., they observe P(O| ∼C). Then, they start the treatment and may check if this base-rate is affected, i.e., they observe P(O| C). In our two experiments, we tried to present a situation that mirrors this natural setting, by observing instances of symptom occurrences on a single individual (additionally, the task was described in second person, to help the participants imagine that they were the patients), and by arranging the information in two phases, one before and one during the treatment.

This choice to split the training session into two phases, P(O| ∼C) and P(O| C), seems to have yielded interesting results. In most similar studies with the traditional task (with the trials arranged in random order), a common finding is that null contingencies are overestimated when the probability of the outcome is high (see reviews in Matute et al., 2015, 2019). Here, Experiment 2 presented a null contingency condition with high chances of remission: in fact, the training in the Infrequent-Control group in which the symptoms were absent in 90% of the trials is almost identical to previous studies that showed strong overestimations of effectiveness, or causal illusions (Blanco et al., 2014; Blanco and Matute, 2019), except for the fact that the trials were separated into two phases, one for P(O| C), and one for P(O| ∼C). This difference seems to have abolished the causal illusion, as Experiment 2 shows clearly that most participants correctly identified the null contingency. We can only speculate as to why this procedural change makes such a big effect on judgments. One possibility is that, by arranging the trials in separate phases, the working memory demands are lower than in the usual experiment, thus making the task easier to solve. A previous study by Willett (2017) tested a related argument. In her experiment, the contingency information was presented in a summarized, pictorial format (depicting faces that represent the cases), rather than trial by trial. The design featured two levels of P(O), high and low, in a null contingency situation. Critically, the pictorial information could be presented in either an “organized” way (which groups together the pictures of faces corresponding to the outcome, on the one hand, and the pictures that represent the no-outcome, on the other hand), or in a “scrambled” way (which intermixed the pictures in a random fashion). We can see a similarity between the scrambled condition and the usual contingency training with intermixed trials, and between the organized condition and our two-phases procedure. This experiment showed that the overestimation of contingency was stronger in the scrambled condition than it was in the organized condition, although the results were only marginally significant. However, one must be cautious when interpreting this evidence, as the information was presented in table format in Willett’s experiment, whereas our experiments used the trial-by-trial format. Future experiments should further investigate the potential sensitivity of causal illusions to cognitive demands on standard trial-by-trial procedures. A second option to interpret the reduced illusion that we found in our Experiment 2 is that, by separating the phases, we are highlighting that outcomes can occur in two different contexts (i.e., in the presence and in the absence of the treatment), hence, implicitly inviting participants to compare them, as in the Δp rule (Allan, 1980). This possibility could be explored in future studies.

The second methodological change from most previously published experiments is the use of a bidirectional scale. As the association between two variables can be either positive or negative, contingency (usually assessed with the Δp index) can take on either positive or negative values, which translates to causally generative scenarios and causally preventive scenarios (Perales et al., 2016). Consequently, the response scale in our experiments was bidirectional, from −100 (the medicine worsens the symptoms) to +100 (the medicine improves the symptoms). Note that most research carried out on contingency learning biases have used the unidirectional scale, from 0 (no effect) to +100 (perfect effectiveness), see, e.g., Matute et al. (2019). The bidirectional scale that we used here has the advantage of correctly capturing the potential range of the contingency and causality values. However, it is also more difficult to understand for some participants. Previous research has suggested that, in general, both types of scale are valid to capture common contingency learning phenomena (see, e.g., Blanco and Matute, 2020, who report the same effects with unidirectional and bidirectional scales).

Finally, in addition to effectiveness judgments and conditional probability estimations, we also collected judgments about the likelihood of using an alternative treatment, aimed at measuring the desire to quit the treatment and look for alternatives. Since the results were the same as those found in the effectiveness ratings, we could conclude that beliefs of effectiveness generalized to this question: participants who saw the disease with high symptom base-rate underestimated the effectiveness of the medicine, and were less willing to adhere to it. Our alternative treatment question contributes, thus, to fill the gap between causal estimations that are typically collected in contingency learning experiments and actual decisions made by patients when dealing with real diseases. The practical implication of our finding is that those patients who underestimate their treatment’s effectiveness are less satisfied, and perhaps are more vulnerable to the offer of alternative options such as pseudomedicines and fraudulent health products (Macfarlane et al., 2020).

### Practical Implications

More generally, we can outline a few implications of our research to clinical practice, although they involve some degree of speculation. Since our procedure is more ecological than the traditional causal learning experiment in certain aspects (order of the information that is presented, observation of only one patient instead of samples…), these experiments are well endowed to inform decisions and insights for real patients using real medicines. The first one is that people use, at best, inefficient methods for assessing effectiveness. Either they are biased by the symptom base-rate directly (as Experiments 1 and 2 initially suggested), or they use proportional change from the symptom base-rate (as Experiment 3 indicated), which is better but still biases the effectiveness assessment, producing lower estimations of effectiveness with larger symptom base-rates. Thus, it is necessary that practitioners watch their patients closely to prevent them from underestimating their treatments’ success, and consequently abandoning the treatment or resorting to pseudomedicine. As mentioned, the patients who are most vulnerable to the effectiveness underestimation are those who initially experience frequent symptoms. Perhaps the misestimation of effectiveness could be reduced if clinicians try to make patients aware that changes in symptom rate proportional to the baseline can be in fact misleading, and provide them with more objective statistics such as absolute differences, when they are available. Previous research suggests that giving this information in frequency format (Bodemer et al., 2014) or pictorial format (Tubau et al., 2019) can greatly improve patients’ comprehension and the chances of communication success. On the other hand, as Experiment 2 shows, the chronological order in which patients usually know the contingency information in natural settings (i.e., first they get to know the symptom base-rate without treatment, then they experience the symptom occurrence rate during the treatment) seems to alleviate other effectiveness estimation problems such as the causal illusion (Matute et al., 2015, 2019) that is more easily observed when the trials are presented in random order. Thus, in this case the natural presentation order works in our favor to prevent the overestimation of effectiveness.

## Data Availability Statement

The experiment materials, the datasets analyzed for this study, and the R scripts to reproduce the tables and figures can be found in the Open Science Framework: https://osf.io/emzbj/.

## Ethics Statement

The studies involving human participants were reviewed and approved by the Ethical Review Board of the University of Deusto (CEUD). The patients/participants provided their written informed consent to participate in this study.

## Author Contributions

FB, MM-F, and HM contributed to the conception and design of the study. MM-F programmed the experiment. FB performed the statistical analysis. FB wrote the first draft of the manuscript. All authors contributed to the manuscript revision, read, and approved the submitted version.

## Conflict of Interest

The authors declare that the research was conducted in the absence of any commercial or financial relationships that could be construed as a potential conflict of interest.

## Footnotes

1 In the typical illusory correlation paradigm, which is often framed in a social context, two groups of people (a minority group and a majority group) possess either of two traits (a common trait and an uncommon trait). Although the two groups have identical trait distributions, it is often concluded that the majority group possesses the common trait to a greater extent than does the minority group (Hamilton and Gifford, 1976). Another paradigm proposed to understand biases in causal learning and illusory correlations is pseudocontingencies (Fiedler et al., 2009; Kutzner et al., 2011), in which people incorrectly use the marginal probabilities of events (e.g., the probability of the cause and the probability of the outcome) as a hint to infer the individual-level contingency, falling prey to an equivalent to the ecological fallacy. In practice, this means that scenarios in which the probability of the cause and the probability of the outcome are skewed in the same direction would produce stronger causal judgments.
